# Novel quantitative trait loci identified for bean rust and the fuscan subspecies of common bacterial blight in an F_2_ tepary bean population

**DOI:** 10.3389/fpls.2026.1924863

**Published:** 2026-10-05

**Authors:** Santos Barrera, Giseli Valentini, Jorge C. Berny Mier y Teran, Tatiana Garcia, Jon Turkus, Marcial A. Pastor-Corrales, Timothy Porch, Carlos A. Urrea

**Affiliations:** 1 Department of Agronomy and Horticulture, University of Nebraska, Lincoln, NE, United States; 2 World Coffee Research, Portland, OR, United States; 3 Department of Plant Science and Plant Pathology, University of Montana, Bozeman, MT, United States; 4 Universidad Nacional de Colombia, Bogotá, Colombia; 5 Soybean Genomics and Improvement Laboratory, USDA-ARS, Beltsville, MD, United States; 6 Tropical Agriculture, Research Station, USDA-ARS, Mayagüez, Puerto Rico

**Keywords:** F2 segregating population, genotyping-by-sequencing (GBS), Phaseolus acutifolius, Phaseolus vulgaris, QTL mapping, Uromyces appendiculatus, Xanthomonas fuscans

## Abstract

Bean rust, caused by *Uromyces appendiculatus*, is one of the most critical diseases affecting common bean (*Phaseolus vulgaris* L.) worldwide. Similarly, common bacterial blight, caused by *Xanthomonas fuscans subsp. fuscans* (Xff) and *Xanthomonas axonopodis pv. phaseoli* (Xap), poses major threats to common bean production globally. While common bean exhibits limited natural resistance to these pathogens, tepary bean (*Phaseolus acutifolius* A. Gray), a closely related species, has shown resistance to both rust and common bacterial blight. This study aimed to identify tepary beans resistant to rust and Xff, to pinpoint genomic regions controlling this resistance. Twenty-three tepary bean genotypes were inoculated with multiple rust races and Xff isolates. Several tepary genotypes exhibited immunity to eight rust races, and a few were also resistant to two isolates of Xff. An F_2_ biparental tepary bean population was developed from one resistant and one susceptible genotype to map quantitative trait loci (QTLs) for resistance to these pathogens. The population was phenotyped by inoculating with two rust races and a mixture of two isolates of Xff. Genotyping by sequencing was employed to genotype the biparental population. A major QTL for the rust pathogen was identified on chromosome Pa10, and a major QTL for common bacterial blight fuscans was identified on chromosome Pa07. Neither of these QTLs have been previously reported in common bean, suggesting that tepary bean possesses different genes controlling the resistance to both pathogens. Both tepary genes should be considered for introgression into common bean through interspecific crossing.

## 1 Introduction

The use of disease-resistant cultivars is by far the most environmentally friendly strategy and the most effective control method for crop disease management. As pathogens evolve, a key component in improving disease-resistant cultivars is the ongoing exploration of new resistant sources and their incorporation into traditional cultivars. The common bean (*Phaseolus vulgaris* L.) is the major legume for direct human consumption (Broughton et al., 2003), and it is a crop of considerable economic importance, including in the United States, where dry beans are produced across a wide range of market classes and growing regions (US Dry Bean Council, 2021). However, global production of common beans is affected by common bean rust caused by *Uromyces appendiculatus* (Pers.) and multiple common bacterial blight pathogens, including *Xanthomonas axonopodis pv. phaseoli* (Xap) and *Xanthomonas fuscans subsp. fuscans* (Xff) (Beebe, 2012). These diseases are among the most devastating for common bean growers worldwide (Tugume et al., 2019). In fact, several of the most resistant cultivars to bacterial blight (Xap), from the Nebraska dry bean breeding program, have shown susceptibility to natural infections of bacterial blight caused by the fuscans subspecies (Xff), indicating that the known resistance to Xap is not reliable when Xff is more prevalent (Urrea, personal communication). It has also been reported that Xff is a more prevalent bacterial blight in certain regions of East Africa (Mkandawire et al., 2004), suggesting that this pathogen could become more widespread globally. In the case of bean rust, resistant cultivars have been developed, but the pathogen is continuously shifting in virulence diversity, and new races of the pathogen are overcoming the resistance in prevalent cultivars (Hurtado-Gonzales et al., 2016; Valentini et al., 2017).

The changes in the prevalence of Xff and the evolving virulence of bean rust underscore the need to explore new resistance sources from related common bean species against both pathogens and to incorporate them into common bean. Tepary bean (*Phaseolus acutifolius* A. Gray) exhibits traits of interest to common bean breeders, including several biotic constraints such as leaf fungal and bacterial diseases (Mhlaba et al., 2018). Resistance to Xap, for instance, has been well researched in tepary beans (Bornowski et al., 2023; Drijfhout and Blok, 1987; Urrea et al., 1999). However, the Xff variant of common bacterial blight, which has been reported as more virulent than Xap (Ekpo and Saettler, 1976; Mutlu et al., 2008) has not been well studied in tepary beans largely due to the lack of differentiation between Xap and Xff. So far, only Opio et al. (1996) reported high levels of resistance in eight tepary beans inoculated with four isolates of Xff. Previous efforts to introduce useful genetic variation from tepary beans into common beans have resulted in the release of interspecific-derived lines that are resistant to Xap (McElroy, 1985; Singh et al., 2001).

There are few studies that explore resistance to common bean rust in tepary beans. Miklas and Stavely (1998) reported seven tepary bean genotypes highly resistant to eight virulent rust races. This resistance in tepary beans was attributed to a single gene with incomplete dominance. Pastor-Corrales et al. (2011) found a domesticated tepary bean with broad resistance to ten Mesoamerican and six Andean races of the bean rust pathogen, and Porch et al. (2022) found rust immunity in several breeding lines, TARS-Tep 22 and TARS-Tep 23.

The genetic control of resistance to these two pathogens has not been fully studied in tepary bean, and resistance to Xff and bean rust has not been introgressed into common bean. However, trait introgression has recently been facilitated by newly developed bridging lines (Barrera et al., 2022), thereby making novel in tepary bean variation more accessible for common bean improvement. To determine the genetic variation associated with resistance to bean rust and Xff in tepary bean, we evaluated the phenotypic response of a set of tepary beans to these two pathogens. After identifying phenotypic variation in tepary bean, we created a biparental F_2_ population to map quantitative trait loci (QTLs) using parents with extreme phenotypes. This is the first QTL mapping study on biotic resistance conducted in tepary bean.

## 2 Materials and methods

### 2.1 Evaluation of tepary bean response to bean rust in 23 cultivated tepary accessions and breeding lines

To determine the resistance response of tepary beans to bean rust, 23 tepary bean accessions and breeding lines were inoculated with six of the most virulent Mesoamerican [13-2 (43), 15-3 (47), 22-6 (49), 31-1 (53), 31-22 (67), and 22-52 (108)], and two Andean races [21-0 (72), 37-1 (84)] of the bean rust pathogen. These 23 genotypes form a defined tepary bean set that we characterized across complementary studies, including a multi-environment evaluation of drought and heat tolerance (Barrera et al., 2024) and the development of interspecific lines for common bean improvement (Barrera et al., 2022). The set spans the domesticated, improved, and wild gene pools of tepary bean: domesticated *P. acutifolius* landraces (G-series accessions from the CIAT *Phaseolus vulgaris* collection), improved USDA-ARS breeding lines (TARS-Tep 22 and TARS-Tep 23), and the wild relative *Phaseolus montanus* Brandegee (syn. *P. parvifolius* Freytag) accession (G40264). This screening set was also used to identify contrasting parents for the biparental QTL population. Inoculation and phenotypic evaluation for preliminary tepary bean resistance screening and for the F_2_ population were identical. Inoculation of the unifoliate (primary) leaves of the bean cultivars was conducted seven days after planting, once the primary leaves were about 2/3 expanded. All plants were inoculated with races 31–22 and 22–52 of *U. appendiculatus*. Races were obtained from the USDA-ARS Bean Project collection at the Beltsville Agricultural Research Center, Beltsville, Maryland.

The inoculum solution was prepared by diluting the frozen urediniospores of *U. appendiculatus* in distilled water containing Tween 20 to obtain a concentration of 2 × 10^4^ urediniospores mL^-1^. Single plants were simultaneously inoculated with races 31–22 and 22–52 of *U. appendiculatus* using a cotton swab to apply the spore solution to the abaxial side of the primary leaves, avoiding contamination between races. For simultaneous inoculation, we cut the tip of the primary leaf and left the other uncut. The cut leaves were inoculated with race 31-22, and the uncut leaves with race 22-52. After inoculation, plants were transferred to a mist chamber (20 ± 1 °C, relative humidity > 95%) and maintained in darkness for approximately 18 hours. After this period, the plants were transferred to the greenhouse and kept for 10–12 days until visible rust symptoms appeared.

The evaluation of the symptoms was performed using a 1 to 6 scale proposed by Stavely et al. (1983), where 1 = no visible rust reaction; 2 = necrotic spots without sporulation (hypersensitive reaction or HR); 2+ = necrotic spots 300–1000 µm (1 mm) in diameter; 2++ = necrotic spots 1–3 mm in diameter; 2+++ = necrotic spots larger than 3 mm in diameter; 3 = tiny sporulating uredinia (rust pustules) less than 300 µm in diameter; 4 = sporulating uredinia 300–500 µm in diameter; 5 = sporulating uredinia 500–800 µm in diameter; and 6 = sporulating uredinia larger than 800 µm in diameter. Plants with scores of 1, 2, and 3 were classified as resistant, whereas plants with scores of 4, 5, and 6 were considered susceptible (**Supplementary Figure S1**).

### 2.2 Phenotypic evaluation of tepary bean response to common bacterial blight, Xff

For the fuscans subspecies of bacterial blight (Xff), 23 tepary bean genotypes were inoculated with a mix of two of the most prevalent strains of Xff (R14 and R19) in Nebraska. The *in vitro* subculture of Xff isolates was completed at the plant pathology lab of the Panhandle and Extension Center of the University of Nebraska-Lincoln in Scottsbluff, NE. A bacterial colony was transferred to a sterile petri dish containing YDCA medium (yeast, calcium carbonate, dextrose, and agar; (Bhutto et al., 2018)) and kept at room temperature for 24 h. The petri dishes were then mailed to the USDA in Beltsville, MD. In Beltsville, after 24 hours of growth, a suspension of each isolate was prepared by transferring the bacteria from each plate into deionized water to a final concentration of 5 × 10^8^ ufc/ml. The bacterial suspensions were adjusted using McFarland standards (Leber, 2016), and then the suspensions of each isolate were combined. The first fully expanded trifoliolate leaf was inoculated on the same plants used for the rust inoculation. Inoculation was conducted using the multiple-needle method reported by Lema Marquez et al. (2007) and Zapata (1985). Three plants from each check and the parents were used as controls for the inoculation. After inoculation, plants were maintained in the greenhouse until disease evaluation. Disease reactions were evaluated at 14 and 21 days after inoculation following the protocol of Lema Marquez et al. (2007), with an additional earlier evaluation at 8 days after inoculation to capture the initial disease response, given the greater aggressiveness of *X. fuscans subsp. fuscans* relative to the *X. axonopodis* isolates used in the original protocol. Disease reactions were recorded by scoring the percentage of diseased area based on a 1–9 scale as described by Lema Marquez et al. (2007) (**Supplementary Figure S2**).

### 2.3 Identification of QTLs associated with the resistance to Xff and bean rust

#### 2.3.1 Plant material

An F_2_ population was developed from a single cross of TARS-Tep 22/G40173A. TARS-Tep 22 (PI 666350) is an improved tepary bean line developed by the USDA-ARS (Porch et al., 2013), while G40173A (doi:10.18730/PFSX8) is a landrace collected in 1987 in Sonora, Mexico. TARS-Tep 22 is resistant to races 31–22 and 22–52 of *U. appendiculatus* and to isolates R14 and R19 of X*. fuscans* subsp. fuscans, while G40173A is susceptible to both races and both isolates. Bean rust reaction was phenotyped across three independent experiments, together comprising 420 F_2_ individuals with a conclusive rust reaction (**Table 1**). Of these, the 293 F_2_ individuals from the first two experiments were additionally inoculated with Xff and subsequently genotyped by genotyping-by-sequencing (GBS); this genotyped subset was used for QTL mapping of both diseases. The third experiment (127 individuals) was phenotyped for rust reaction only and was not genotyped or used in QTL mapping. In each experiment, the rust pathogen was inoculated on the primary leaves and the common bacterial blight pathogen (where applicable) was inoculated on the first trifoliate leaf; parents and common bean checks Pinto 114, Aurora (Ur-3), Early Gallatin (Ur-4), and Golden Gate Wax (Ur-6) were used as controls of successful inoculation in each experiment.

**Table 1:** Segregation for reaction to bean rust in the F2 population TARS-Tep 22 (R)/G40173A (S), inoculated simultaneously with races 31–22 and 22–52 of *Uromyces appendiculatus* under greenhouse conditions.

Race	Inoculation batch	Immune (1)	Resistant, TP (2-3)	Susceptible (4-6)	Total	χ²	P
31-22	Batch 1	42	66	41	149	1.95	0.38
	Batch 2	40	72	32	144	0.89	0.64
	**Genotyped subset (Batch 1 + 2)**	**82**	**138**	**73**	**293**	**1.54**	**0.46**
	Batch 3	32	65	30	127	0.13	0.94
	**Total observed**	**114**	**203**	**103**	**420**	**1.04**	**0.59**
	*Expected (1:2:1), genotyped subset*	*73.25*	*146.5*	*73.25*	*293*	*-*	*-*
	*Expected (1:2:1), all experiments*	*105*	*210*	*105*	*420*	*-*	*-*
22-52	Batch 1	41	66	42	149	1.95	0.38
	Batch 2	39	72	33	144	0.5	0.78
	**Genotyped subset (Batch 1 + 2)**	**80**	**138**	**75**	**293**	**1.16**	**0.56**
	Batch 3	32	65	30	127	0.13	0.94
	**Total observed**	**112**	**203**	**105**	**420**	**0.7**	**0.7**
	*Expected (1:2:1), genotyped subset*	*73.25*	*146.5*	*73.25*	*293*	*-*	*-*
	*Expected (1:2:1), all experiments*	*105*	*210*	*105*	*420*	*-*	*-*

Bold values are the pooled rows. The genotyped subset combines inoculation batches 1 and 2 (n = 293) and is the population used for QTL mapping.

#### 2.3.2 Phenotypic evaluation of the bean rust disease

The phenotypic evaluations were conducted at the Soybean Genomics and Improvement Laboratory of the USDA-ARS Beltsville Agricultural Research Center in Beltsville, Maryland, USA, across three independent inoculation batches. Inoculation of the F_2_ plants followed exactly the simultaneous dual-race procedure described in Section 2.1: on each plant, the tip of one primary leaf was cut, and that leaf was inoculated with race 31-22, while the opposite, uncut primary leaf was inoculated with race 22-52, so that every F_2_ individual was scored for both races. Batches 1 and 2 comprised 149 and 144 F2 plants, respectively. All 293 F_2_ individuals from batches 1 and 2 were additionally inoculated with Xff and genotyped by GBS for joint QTL mapping. Batch 3 phenotyped a further 127 F_2_ individuals for rust reaction only; these plants were not inoculated with Xff and were not genotyped and are therefore not part of the QTL-mapping population but are included in the full rust segregation summary (**Table 1**). Plants were maintained in 12.7-cm diameter pots under greenhouse conditions at 20 ± 5 ^∘^C. The evaluation of the symptoms was performed using a 1 to 6 scale proposed by (Stavely et al., 1983), where 1 = no visible rust reaction; 2 = necrotic spots without sporulation (hypersensitive reaction or HR); 2+ = necrotic spots 300–1000 μm (1 mm) in diameter; 2++ = necrotic spots 1–3 mm in diameter; 2+++ = necrotic spots larger than 3 mm in diameter; 3 = tiny sporulating uredinia (rust pustules) less than 300 μm in diameter; 4 = sporulating uredinia 300–500 μm in diameter; 5 = sporulating uredinia 500–800 μm in diameter; and 6 = sporulating uredinia larger than 800 μm in diameter. Plants with scores of 1, 2, and 3 were classified as resistant, whereas plants with scores of 4, 5, and 6 were considered susceptible.

#### 2.3.3 Phenotypic evaluation of the common bacterial blight disease

The 293 F_2_ plants from the single cross of TARS-Tep 22(R)/G40173A(S) that were inoculated with the rust pathogen were then inoculated with the common bacterial blight fuscans pathogen (*X. fuscans subsp. fuscans*). Inoculation was conducted using the multiple-needle method reported by Lema Marquez et al. (2007) and Zapata (1985). Three plants from each check and the parents were used as controls for the inoculation. After inoculation, plants were maintained in the greenhouse until disease evaluation. Disease reactions were evaluated at 8, 14, and 21 days after inoculation by scoring the percentage of diseased area based on a 1–9 scale as described by Lema Marquez et al. (2007).

### 2.4 DNA extraction and sequencing

Genomic DNA was extracted from 293 F_2_ plants and 2 parental lines using the MagMax™ Plant DNA Isolation kit (A32549) (ThermoFisher Scientific) following the instructions of the manufacturer. The integrity of the DNA was inspected using agarose gel electrophoresis prepared with 35 mg agarose, 35 mL 1xTBE (Tris–borate–ethylenediaminetetraacetic acid) and dyed with 0.75 µL ethidium bromide. DNA was quantified with the Invitrogen Quant-iT dsDNA Broad Range Quantitation assay kit (Q33120) (ThermoFisher Scientific) and a CLARIOstar^®^ Plus plate reader (BMG Labtech). Linear regression standard curves were generated using MARS software (BMG Labtech). DNA was normalized to 20 ng/µL prior to sequencing.

Genotyping-by-sequencing (GBS) of 295 DNA samples was carried out at the Genome Center of the University of California, Davis, where DNA Libraries were prepared and sequenced using a HiSeq 4000 SR100SE instrument (Illumina Inc., San Diego, CA). It has been established that the CviAII restriction enzyme is more suitable for GBS in common beans than the traditional ApeKI enzyme (Ariani et al., 2016). For this reason, given the close genetic relationship between tepary and common beans, the CviAII restriction enzyme was used for GBS in this study.

### 2.5 Read quality control, read alignment, SNP calling, filtering, imputation, and error correction

DNA sequence reads were demultiplexed from 95-pooled sample libraries using the ‘Demultiplex’ option in the Next Generation Sequencing Experience Platform (NGSEP) (Duitama et al., 2014; Tello et al., 2019), which were then checked for quality using the FastQC program (Andrews, 2010). Illumina Adaptors and low-quality reads were trimmed using the Trimmomatic program (Bolger et al., 2014). The reads were aligned to the Frijol Bayo reference genome of tepary bean (*P. acutifolius* v1.0 = G40001) (Moghaddam et al., 2021) using the ‘ReadsAligner’ option in the NGSEP platform and the ‘Sortsam’ option of the Picard toolkit (“Picard Toolkit.” 2019. Broad Institute, GitHub Repository, http://broadinstitute.github.io/picard/; Broad Institute). Single Nucleotide Polymorphisms (SNPs) were called with the ‘MultisampleVariantsDetector’ option in the NGSEP platform. Multiple filtering criteria (minor allele frequency [minMAF 0.25], parental alleles, missingness [75%], and depth coverage [7 reads]) were performed with the NGSEP platform and the TASSEL v5 GUI program, following the methodology proposed by Furuta et al. (2017). After filtering, a variant call file (vcf) with a resulting matrix of 3,127 SNPs was converted to the ABH format using the ‘GenosToABHPlugin’ from TASSEL v.5.0 (Bradbury et al., 2007). This matrix was then imputed and error-corrected in R (R Core Team, 2025) using the R package ABHGenotypeR (Furuta et al., 2017), yielding 4,990 SNPs.

### 2.6 QTL mapping

A genetic linkage map was developed with the R package “asMAP” (Taylor and Butler, 2017), filtering out genotypes/markers with more than 20% missing data, with segregation distortion (p<0.001), and co-located markers. After filtering, a data set of 1,004 SNPs was used to construct the genetic map. The marker position and its recombination estimates were obtained with the Kosambi mapping function (Kosambi, 1944).

The mapping of QTLs was carried out with rqtl2 (Broman et al., 2019) with linear mixed models and with “leave one chromosome out” as a covariate, which uses a kinship matrix calculated from all the other chromosomes. The LOD significance threshold was estimated from 1,000 permutations at a genome-wide significance level of α = 0.05 (Churchill and Doerge, 1994). The support interval for each QTL was estimated as the 95% Bayesian credible interval. Allelic effects were estimated directly through the linear mixed models implemented within the R/qtl2 package (Broman et al., 2019), which calculates the additive and dominance effect coefficients for each genotypic class (AA, AB, BB) at the marker located nearest to the QTL peak.

## 3 Results

To identify the genetic variation associated with resistance to bean rust in tepary beans, a set of tepary bean genotypes was inoculated with virulent races of the bean rust pathogen. We found that all control common bean cultivars were susceptible to one or more of the races of *U. appendiculatus* (**Supplementary Table 1**). Conversely, four domesticated tepary bean accessions (G40142, G40148, G40161, and G40237A) and two improved lines (TARS-Tep 22 and TARS-Tep 23) were immune to all eight races of the bean rust pathogen (**Supplementary Table 1**). The immune reaction, with no visible symptoms, observed in six tepary beans is not known to occur in common beans. Moreover, two domesticated tepary bean accessions (G40274 and G40279) and one wild *Phaseolus montanus* Brandegee (syn. *P. parvifolius* Freytag) accession (G40264) was resistant to all eight races. These accessions exhibited either hypersensitive reactions (HR) with necrotic spots without sporulation or tiny sporulating pustules (TP) (**Supplementary Table 1**). The HR and TP reactions are consistently cited as the resistant reaction in common beans. However, the immune reaction with no visible symptoms suggests the presence of new and unique rust resistance genes. Previous reports of resistance to the bean rust pathogen in tepary beans by Miklas and Stavely (1998) indicated seven tepary genotypes highly resistant to eight bean rust races. They also showed that the resistance reaction was due to a single gene with incomplete dominance. Similarly, Pastor-Corrales et al. (2011) found one tepary bean accession (G40022) highly resistant to ten Mesoamerican and six Andean races of the bean rust pathogen, and Porch et al. (2022) found TARS-Tep 22 and TARS-Tep 23 to be immune to rust, thus confirming the results of this study.

To identify the genetic variation associated with resistance to bacterial blight fuscans (Xff) in tepary beans, a set of tepary bean genotypes was inoculated with virulent strains of the bacterial blight Xff pathogen. We found that all common beans inoculated in this study were completely susceptible to both strains of *X. fuscans subsp. fuscans* (**Supplementary Table 2**). We also found that only one improved tepary bean line (TARS-Tep 22) was consistently resistant throughout the three evaluations (**Supplementary Table 2**). The same result was confirmed by a second inoculation which demonstrated the resistant reaction (**Supplementary Table 2**). These results indicate that resistance to Xff in tepary beans varies by genotype. It also indicates that resistance to Xff maybe less represented in tepary bean than resistance to bean rust.

### 3.1 Identification of QTLs associated with the resistance to bean rust and common bacterial blight fuscans (Xff) in an F_2_ biparental population of tepary beans

A binary classification system was used to categorize plant responses to bean rust inoculation. Plants with resistance scores of 1, 2, and 3 were classified as resistant, whereas those with scores of 4, 5, and 6 were classified as susceptible. Segregation analysis of the F2 population for reaction to race 31–22 revealed a 1:2:1 ratio (**Table 1**), indicating that a single gene controls the resistance trait in this population. This incomplete dominance pattern, in which heterozygous individuals exhibit partial gene expression, was confirmed by the same 1:2:1 ratio observed in the allele distribution matrix constructed from SNP data (**Figure 1**). Reaction to race 22–52 was scored on the same plants and was concordant with the reaction to race 31-22; the counts presented in **Table 1** are therefore those for race 31–22 and are representative of both races. In contrast, the phenotypic evaluation of Xff was recorded as a continuous variable, precluding calculation of segregation ratios for this trait.

**Figure 1:**
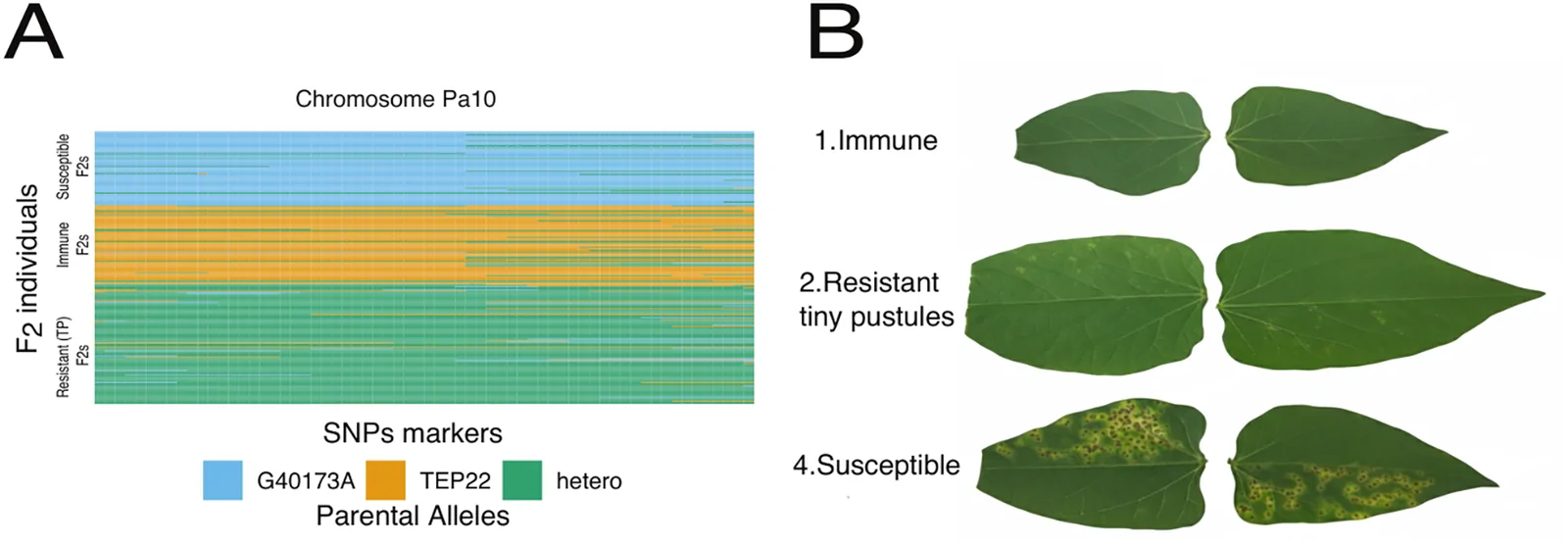
Graphical representation of the F_2_ phenotypes (rust reaction). **(A)** Parental alleles of Chromosome 10 assigned on Tassel 5.0 and plotted using the ABHgenotypeR package. **(B)** disease severity phenotypes: 1. Immune (healthy leaves), 2. Resistant (leaves with tiny pustules), and 4. Susceptible (leaves with numerous large pustules).

### 3.2 QTL mapping

After filtering, 1,004 markers were used to develop the genetic map. It comprised 11 genetic groups, numbered according to the tepary bean reference genome (**Table 2**). The map length was 1349.4 centimorgans (cM), with an average of 91 markers per chromosome. Marker density varied among chromosomes. The lower marker numbers on Pa05 (4 markers) and Pa11 (16 markers) reflect low polymorphism between the two parental lines in these specific regions, likely due to shared parentage. Genome coverage, as reported in **Table 2**, is the proportion of each chromosome spanned by the interval between its first and last mapped marker. It is therefore set by the physical position of the two terminal markers and is independent of how many markers fall between them; coverage and marker density describe different properties of a linkage group and need not vary together. Across all 11 chromosomes, coverage ranged from 49.5% on Pa05 to 89.0% on Pa02 (**Table 2**). Coverage did not scale directly with marker number: Pa11 reached 83.3% coverage with only 16 markers, exceeding Pa08 (82.5%), which carries the most markers on the map (205), and Pa02 gave the highest coverage of the map (89.0%) with 56 markers, well above Pa04 (81.3%) with 168. Conversely, Pa10 is the second densest chromosome (152 markers) yet has the third lowest coverage (73.0%). Average marker spacing across the whole map was 1.4 cM (1,004 markers over 1,349.4 cM), and the largest gap between adjacent markers was 26.6 cM, on chromosome Pa02 (**Table 2**).

**Table 2:** Distribution of SNP markers across the 11 linkage groups of the tepary bean genetic map.

Chromosome	Numer of SNPs	Length	Average spacing (cM)	Maximum spacing (cM)	Physical range (bp)		Physical length (bp)	Genome coverage (%)
		cM	cM	cM	bp	bp		
1	133	222.1	1.7	21.2	3177305	53076626	56952050	87.62
2	56	130.5	2.4	26.6	3870652	44070951	40664972	98.86
3	82	159.6	2	21.3	1233679	39850686	40304639	95.81
4	168	130.7	0.8	11.9	6717450	54844908	59168269	81.34
5	4	22.2	7.4	19.9	3063514	21377336	33276795	55.03
6	46	79.2	1.8	14	6227862	23746481	32545300	53.83
7	73	117.5	1.6	26	3478222	39804187	40051661	90.70
8	205	233.2	1.1	21.8	7273897	52833063	55222839	82.50
9	69	92.7	1.4	11.2	923044	32540812	35673976	88.63
10	152	113.7	0.8	3.6	6566010	36081458	36371146	81.15
11	16	47.9	3.2	18	3612722	46996351	46888121	92.53
**Overall**	**1004**	**1349.4**	**1.4**	**26.6**				

Bold values in the final row are the map-wide summaries across all 11 linkage groups.

To find the genomic regions associated with resistance to Xff in tepary bean, a Quantitative Trait Loci (QTL) study was carried out in an F_2_ tepary bean biparental population. We identified a major QTL associated with resistance to Xff on Chr Pa07 (**Figure 2**). This exceeded the estimated LOD threshold (LOD = 3.6). All three phenotypic evaluations showed the same peak (**Figure 2**). However, the evaluation conducted 21 days after the inoculation (3rd evaluation) showed the highest LOD score located at the 43 centimorgan (cM) position, with a LOD score of 35.4 and a percentage of explained variation of 43% (**Table 3**). The confidence interval for this QTL was from 5.86 to 21.94 megabase pairs (Mbp). The resistance source allele was conferred by TARS-Tep 22 (allele B) (**Figure 3**). The resistant allele had an effect of reducing the Xff phenotypic score by 1.47, from the mean of the population to a level of 2.51, on a scale of 1 (resistant) to 9 (susceptible), while the heterozygous allele had an intermediate score (**Table 3**).

**Figure 2:**
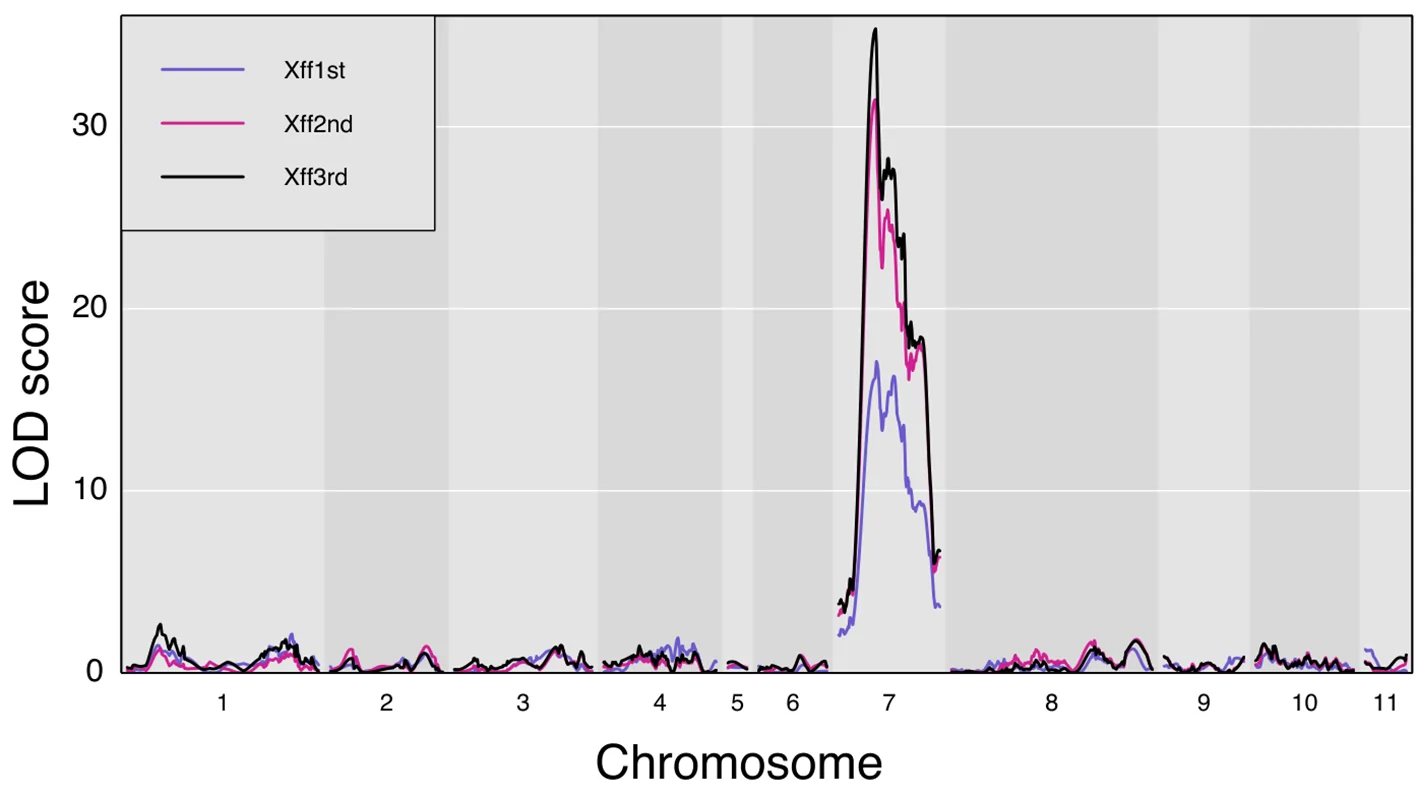
QTL mapping for common bacterial X. fuscans subsp. fuscans (Xff) evaluated in an F_2_ tepary bean population. LOD: Logarithm of odds score. Three phenotypic evaluations were performed: 1st: 8 days after inoculation; 2nd: 14 days after inoculation; and 3rd: 21 days after inoculation.

**Table 3:** QTLs found for Xff and bean rust, and their genetic and physical location, coefficient effects, and percent of explained variation (PEV).

				Confidence interval								
				Genetic		Physical		Estimated coefficients				
Trait	Chr	Position	LOD	Low	Hi	Low	Hi	AA	AB	BB	Intercept	PEV
Xff	7	43	35.4	16.584	45.2114	5865569	2.2E+07	2.1	-0.63	-1.47	3.98	43%
Rust	10	92	171	91.008	92.5447	2.8E+07	2.8E+07	0.59	-0.28	-0.32	0.32	93%

**Figure 3:**
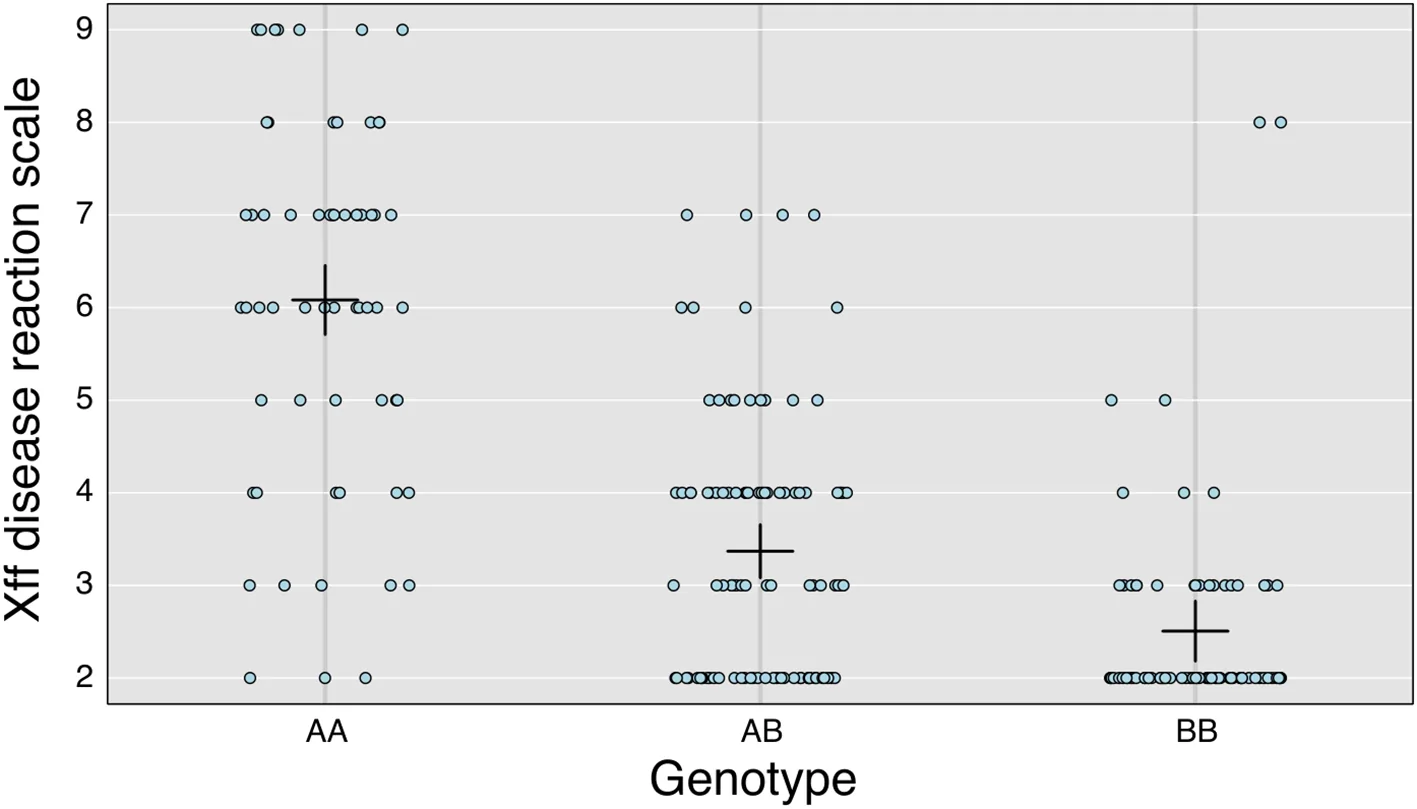
Allele effect of the QTL located on chromosome Pa07 for Xff. The allele AA corresponds to G40173A, and allele BB corresponds to TARS-Tep 22.

To find the genomic regions associated with resistance to bean rust in tepary beans, a quantitative trait loci (QTL) study was conducted using the same F_2_ tepary bean biparental population previously employed for the Xff study. A major QTL was detected on Chromosome Pa10 associated with a resistance reaction to the bean rust pathogen. This QTL showed a LOD likelihood ratio above the estimated threshold (LOD threshold=3.45) (**Figure 4**). Rust resistance was evaluated with two races of *U. appendiculatus*, race 31-22, and race 22-52. The QTL showed similar peaks for both races, and the results for race 31 were used for further analyses.

**Figure 4:**
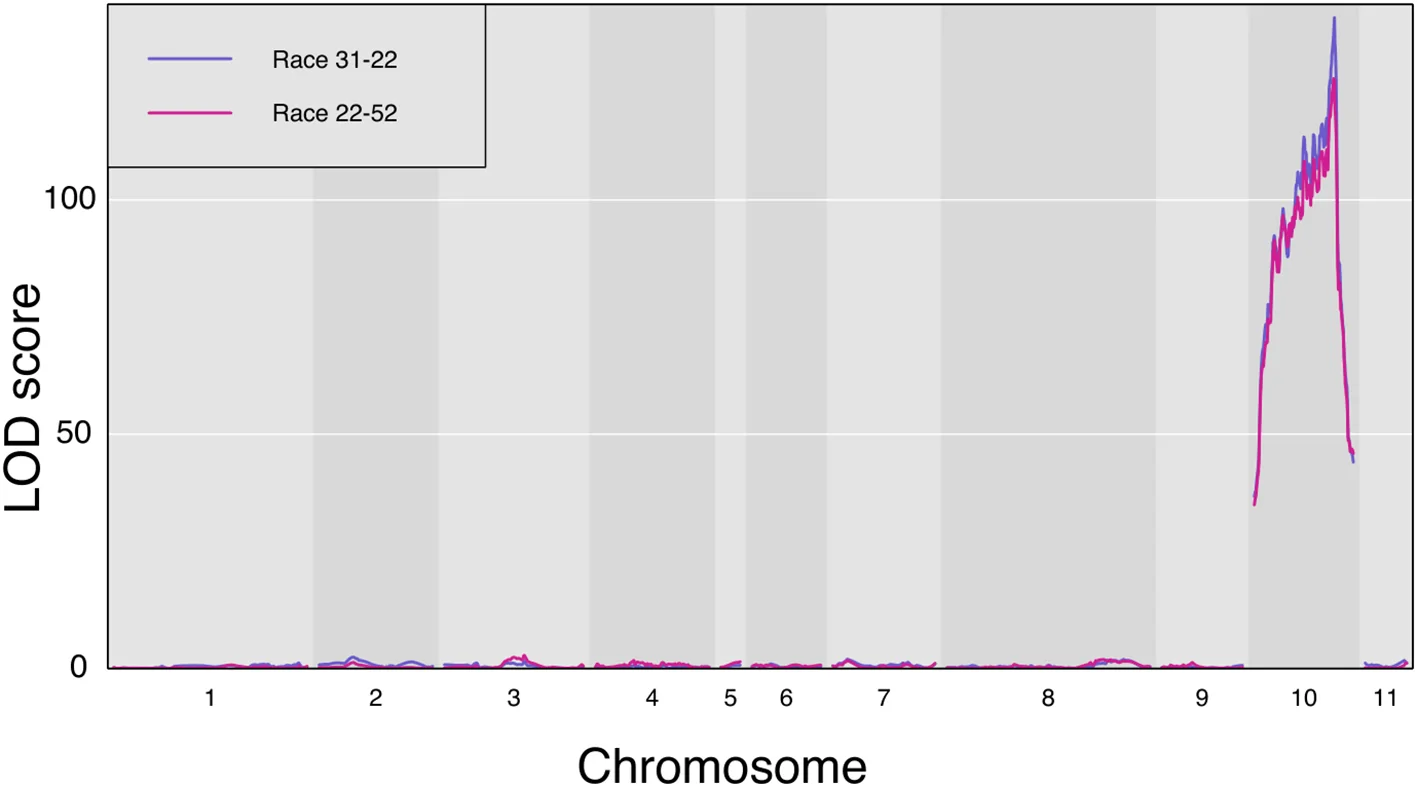
QTL mapping for bean rust U. appendiculatus evaluated in an F_2_ tepary bean population. LOD: Logarithm of odds score. The two inoculated races (31–22 and 22-52) are plotted.

The QTL peak mapped to 92 cM with an exceptionally high LOD score of 171, explaining 93% of the phenotypic variation and indicating near-complete genetic control of the resistance trait (**Table 3**). The credible interval spanned 27.98–28.42 Mbp (1.54 cM), delimiting a narrow target region for candidate gene identification. Among the genes annotated within this interval, two are of particular interest. *Phacu.CVR.010G106600*, located approximately 154 kb~ from the LOD maximum, encodes a protein combining S-locus glycoprotein, bulb-type lectin, and serine/threonine/tyrosine kinase domains—a domain architecture characteristic of S-locus lectin receptor-like kinases, a well-documented class of plant pattern-recognition receptors. *Phacu.CVR.010G104700*, located approximately 281 kb~ from the LOD maximum, encodes a glycoside hydrolase family protein; this enzyme family includes chitinases and glucanases active against fungal cell walls, making it plausibly relevant to resistance against the rust pathogen. Both genes are proposed here as positional candidates only. The present data provide no sequence or expression comparison between the two parents at either locus, and functional validation will be required before either can be linked to the resistance reaction.

Allelic analysis revealed that the resistance allele originated from the TARS-Tep 22 parent (allele B), consistent with its known resistance phenotype (**Figure 5**). Genotype-phenotype associations showed clear incomplete dominance: most of the homozygous resistant individuals (BB) displayed complete immunity, with only a few exceptions that showed tiny pustule-resistant reactions, heterozygotes (AB) exhibited resistance characterized by small pustule formation, while homozygous susceptible individuals (AA) carrying the G40173A allele were fully susceptible to infection (**Figure 5**).

**Figure 5:**
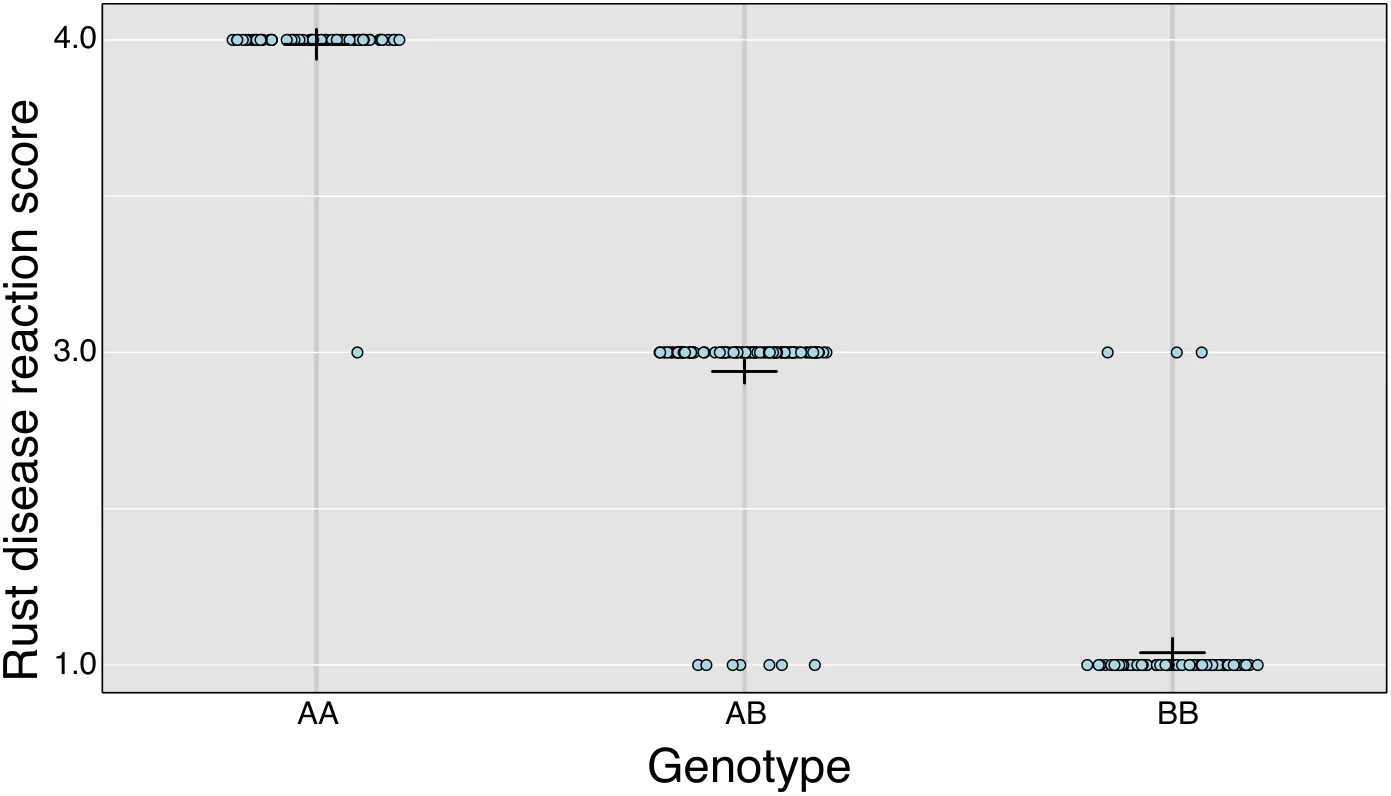
Allele effect of the QTL located on chromosome Pa10 for bean rust. The allele AA corresponds to G40173A, and allele BB corresponds to TEP22.

## 4 Discussion

This study demonstrates the presence of unique rust resistance reactions in tepary bean (**Supplementary Table 1**). Additionally, we corroborate that resistance to bacterial blight (Xff) is present in tepary bean (**Supplementary Table 2**). Most importantly, we described the first disease-resistance QTL mapping conducted in tepary beans. The identification of a major QTL for Xff on chromosome Pa07 in tepary bean indicates the presence of a novel resistant genetic source that has not been found in common bean, mainly because Xff has not been studied as much as the traditional common bacterial blight (CBB) (*X. axonopodis pv. phaseoli*). To our knowledge, this is also the first study to map QTLs for two distinct diseases within the same tepary bean F_2_ population, allowing both resistance loci to be characterized under a single, consistent experimental and analytical framework.

Previous mapping studies have identified 14 dominant rust resistance genes in common bean, of which ten have been genetically mapped across six chromosomes. Specifically, the genes are located as follows: *Ur-9* on chromosome Pv01; *Ur-5* and *Ur-14* on Pv04; *Ur-4* on Pv06; *Ur-12* on Pv07; *Ur-13* on Pv08; and *Ur-3*, *Ur-6*, *Ur-7*, and *Ur-11* on Pv11 (Leitao et al., 2023). Notably, no mapped *Ur* gene has been assigned to Pv10, which corresponds to Pa10 in the largely collinear relationship between the two genomes (Moghaddam et al., 2021). This observation highlights the novelty of the QTL identified in our study. Additionally, major and minor QTLs have been identified for resistance to CBB in common bean, and several key molecular markers are now widely used in breeding programs. For instance, a resistant QTL was reported to be associated with resistance to CBB in common beans on Pv10 (Miklas et al., 2003), which resulted in the development of a SCAR molecular marker (SAP6) widely used in molecular-assisted selection (MAS) (Jung et al., 1999; Viteri et al., 2014). Also, two molecular markers, BC420 located on Pv06 (Yu et al., 2000) and SU91 located on Pv08 (Pedraza et al., 1997), are commonly used for MAS in bean breeding programs. The resistant QTLs associated with these two markers were reported in XAN159, an interspecific common-tepary bean line (Pedraza et al., 1997). Miklas et al. (2006) mentioned the presence of two QTLs associated with resistance to CBB on chromosomes Pv05 and Pv07, but no molecular markers associated with these QTLs have been developed. A recent genome-wide association study (GWAS) conducted on a tepary bean diversity panel identified multiple genomic regions linked to CBB resistance (Bornowski et al., 2023). Notably, this study identified a SNP (S07_14152143) that falls within the confidence interval of our current findings. These convergent results suggest that the Pa07 locus may confer dual resistance to both Xff and Xap pathogens in tepary bean. Because Bornowski et al. (2023) evaluated resistance to Xap in an independent, unrelated diversity panel using a genome-wide association approach, while the present study evaluated resistance to Xff using biparental linkage mapping, the convergence of both studies on the same Pa07 region constitutes independent support for this locus rather than a coincidental overlap. Also, QTLs associated with resistance to Xff have not been identified in common beans, underscoring the importance of our finding and the urgent need to continue this work and fine-mapping the underlying genes to introgress the resistance to Xff into common beans.

Here, we report the first genetic mapping of resistance to bean rust in tepary bean. We confirmed the presence of an incomplete-dominant gene that confers high levels of resistance to bean rust in tepary beans (**Table 1**), originally identified by Miklas and Stavely (1998). Mapping efforts for bean rust (*Uromyces appendiculatus*) have identified race-specific Ur genes and closely linked molecular markers in common beans. Early marker-tagging studies using bulked segregant analysis and RAPD identified markers linked to single dominant rust-resistance genes in common bean crosses (Park et al., 1997). More recent high-resolution mapping studies have revealed a linkage between genes conferring resistance to rust, anthracnose, and angular leaf spot diseases, providing opportunities for multi-disease resistance breeding (Valentini et al., 2017). The major resistance gene located on chromosome Pa10 (**Figure 4**) indicates the presence of a novel resistance not previously described in common bean (Valentini et al., 2017). This finding is particularly significant as it represents the first genetic mapping of rust resistance specifically in tepary bean, potentially providing a new source of resistance genes that could be transferred to common bean. Transferring the rust-resistant gene found here to the common bean is an essential future objective. The development of molecular markers closely linked to this rust resistance gene will be crucial for efficient marker-assisted introgression programs. However, given the comparatively wide credible interval of the Pa07 QTL (28.63 cM; 16.07 Mb), further fine-mapping, potentially in a recombinant inbred population, will be needed to narrow this region and refine the candidate gene search for Xff resistance.

The recent release of the tepary bean reference genome (Moghaddam et al., 2021) has created opportunities to identify quantitative trait loci (QTLs) linked to resistance genes associated with biotic constraints. The identification of QTLs in tepary beans could be useful for molecular-marker-assisted selection in tepary beans as well as gene introgression into common beans. The availability of high-quality genomic resources allows for the development of high-density SNP arrays and the identification of candidate genes underlying QTL regions.

Two limitations of the present study should be stated explicitly. First, QTL detection and effect estimation rest on 293 genotyped F2 individuals. This is ample for the major-effect loci reported here, which explained 43% and 93% of the phenotypic variation, but it provides limited power to detect minor-effect QTL. Consequently, additional loci with smaller effects that contribute to either trait may have gone undetected. Second, an F2 population carries only a single round of meiotic recombination, which places a hard ceiling on mapping resolution. This limitation is particularly evident in the Xff QTL, whose 95% credible interval spans 28.63 cM and 16.07 Mb, a region that includes far too many annotated genes for effective candidate prioritization. In contrast, the rust QTL is narrowed down to a much smaller interval (1.54 cM; 0.44 Mb), but this narrow range is due to the substantial size of its effect, not because of any increased recombination in the population. To improve our findings, advancing this cross to recombinant inbred lines and increasing the population size will be necessary to further narrow the Pa07 interval and validate the candidate genes proposed for Pa10.

## Data Availability

The raw genotyping-by-sequencing reads (Illumina HiSeq 4000, single-end 100 bp; 301 runs from 293 F2 individuals and the parents TARS-Tep 22 and G40173A) have been deposited in the NCBI Sequence Read Archive under BioProject accession PRJNA1535473 (https://www.ncbi.nlm.nih.gov/bioproject/PRJNA1535473).

## Supplementary material

Supplementary Table 1 Reaction of tepary and common bean genotypes to six Mesoamerican and two Andean races of *U. appendiculatus*.

Supplementary Table 2 Reaction of tepary and common bean genotypes to a mix of two isolates of *X. fuscans* subsp. *fuscans*.

Supplementary Figure 1 Representative reactions of the bean rust (*U. appendiculatus*) 1–6 rating scale (Stavely et al., 1983). (A) grade 1, immune; (B) grade 2 (2+, 2++), necrotic spots without sporulation (HR); (C) grade 3, tiny sporulating uredinia <300 μm (SEM inset); (D) grades 4–6, sporulating uredinia of 300–500, 500–800, and >800 μm (SEM inset). Plants scoring 1–3 were classified as resistant and 4–6 as susceptible.

Supplementary Figure 2 Representative reactions of the common bacterial blight (Xff) 1–9 severity scale (Lema Marquez et al., 2007): 1 = no visible symptoms; 3 = isolated, small chlorotic zones around necrotic lesions (<25% of the inoculated area); 5 = chlorotic zones joined within <50% of the inoculated area; 7 = complete chlorosis of the inoculated area extending beyond it; 9 = severely diseased, with large chlorotic lesions also in uninoculated areas; even values represent intermediate reactions.

## References

[B1] Andrews S. (2010). A quality control tool for high throughput sequence data. Available online at: https://www.bioinformatics.babraham.ac.uk/projects/fastqc/ (Accessed August 21, 2021).

[B2] Ariani A. Berny Mier y Teran J. C. Gepts P. (2016). Genome-wide identification of snps and copy number variation in common bean (*phaseolus vulgaris* l.) using genotyping-by-sequencing (gbs). Mol. Breed. 36, 1–11. doi: 10.1007/s11032-016-0512-930311153

[B3] Barrera S. Berny Mier y Teran J. C. Aparicio J. Diaz J. Leon R. Beebe S. . (2024). Identification of drought and heat tolerant tepary beans in a multi-environment trial study. Crop Sci. 64, 3399–3416. doi: 10.1002/csc2.2135442587375

[B4] Barrera S. Berny Mier y Teran J. C. Lobaton J. D. Escobar R. Gepts P. Beebe S. . (2022). Large genomic introgression blocks of *phaseolus parvifolius* freytag bean into the common bean enhance the crossability between tepary and common beans. Plant Direct 6, e470. doi: 10.1002/pld3.470 36523608 PMC9745863

[B5] Beebe S. E. (2012). Common bean breeding in the tropics. Plant Breed. Rev. 36, 357–426. doi: 10.1002/9781118358566.ch542587375

[B6] Bhutto S. H. Tarique J. A. Hajano J. U. Syed R. N. Jatoi G. H. Ismail M. . (2018). Some biochemical characterization against *xanthomonas oryzae pv. oryzae* cause bacterial leaf blight of rice. Pakistan J. Biotechnol. 15, 429–434.

[B7] Bolger A. M. Lohse M. Usadel B. (2014). Trimmomatic: A flexible trimmer for illumina sequence data. Bioinformatics 30, 2114–2120. doi: 10.1093/bioinformatics/btu170 24695404 PMC4103590

[B8] Bornowski N. Hart J. P. Palacios A. V. Ogg B. Brick M. A. Hamilton J. P. . (2023). Genetic variation in a tepary bean (*phaseolus acutifolius* a. gray) diversity panel reveals loci associated with biotic stress resistance. Plant Genome 16, e20363. doi: 10.1002/tpg2.20363 37332263 PMC12807419

[B9] Bradbury P. J. Zhang Z. Kroon D. E. Casstevens T. M. Ramdoss Y. Buckler E. S. (2007). Tassel: Software for association mapping of complex traits in diverse samples. Bioinformatics 23, 2633–2635. doi: 10.1093/bioinformatics/btm308 17586829

[B10] Broman K. W. Gatti D. M. Simecek P. Furlotte N. A. Prins P. Sen S. . (2019). R/qtl2: Software for mapping quantitative trait loci with high-dimensional data and multiparent populations. Genetics 211, 495–502. doi: 10.1534/genetics.118.301595 30591514 PMC6366910

[B11] Broughton W. J. Hernandez G. Blair M. Beebe S. Gepts P. Vanderleyden J. (2003). Beans (*phaseolus* spp.) - model food legumes. Plant Soil 252, 55–128. doi: 10.1023/A:1024146710611

[B12] Churchill G. A. Doerge R. W. (1994). Empirical threshold values for quantitative trait mapping. Genetics 138 (3), 963–971. doi: 10.1093/genetics/138.3.963 PMC1206241 7851788

[B13] Drijfhout E. Blok W. J. (1987). Inheritance of resistance to *xanthomonas campestris pv phaseoli* in tepary bean (*phaseolus acutifolius*). Euphytica 36, 803–813. doi: 10.1007/BF0005186330311153

[B14] Duitama J. Quintero J. C. Cruz D. F. Quintero C. Hubmann G. Foulquié-Moreno M. R. . (2014). An integrated framework for discovery and genotyping of genomic variants from high-throughput sequencing experiments. Nucleic Acids Res. 42. doi: 10.1093/nar/gkt1381 24413664 PMC3973327

[B15] Ekpo E. J. Saettler A. W. (1976). Pathogenic variation in *xanthomonas phaseoli* and *xanthomonas phaseoli var. fuscans*. Plant Dis. Rep. 60, 80–83.

[B16] Furuta T. Ashikari M. Jena K. K. Doi K. Reuscher S. (2017). Adapting genotyping-by-sequencing for rice f2 populations. G3: Genes Genomes Genet. 7, 881–893. doi: 10.1534/g3.116.038190 28082325 PMC5345719

[B17] Hurtado-Gonzales O. P. Valentini G. Gilio T. A. S. Martins A. M. Song Q. Pastor-Corrales M. A. (2016). Fine mapping of ur-3, a historically important rust resistance locus in common bean. G3 (Bethesda Md.) 7, 557–569. doi: 10.1534/g3.116.036061 28031244 PMC5295601

[B18] Jung G. Skroch P. Nienhuis J. Coyne D. Arnaud-Santana E. Ariyarathne H. . (1999). Confirmation of qtl associated with common bacterial blight resistance in four different genetic backgrounds in common bean. Crop Sci. 39, 1448–1455. doi: 10.2135/cropsci1999.3951448x

[B19] Kosambi D. D. (1944). The estimation of map distances from recombination values (New Delhi: Springer). doi: 10.1111/j.1469-1809.1943.tb02321.x

[B20] Leber A. L. (2016). doi: 10.1128/9781555818814.ch5.20.1

[B21] Leitao S. T. Rubiales D. Vaz Patto M. C. (2023). Identification of novel sources of partial and incomplete hypersensitive resistance to rust and associated genomic regions in common bean. BMC Plant Biol. 23, 610. doi: 10.1186/s12870-023-04619-8 38041043 PMC10691055

[B22] Lema Marquez M. Terán H. Singh S. P. (2007). Selecting common bean with genes of different evolutionary origins for resistance to *xanthomonas campestris pv. phaseoli*. Crop Sci. 47, 1367–1374. doi: 10.2135/cropsci2006.12.0769

[B23] McElroy J. B. (1985). Breeding dry beans, Phaseolus vulgaris L. for common bacterial blight resistance derived from Phaseolus acutifolius A. Gray (Itaca, NY: Cornell University).

[B24] Mhlaba Z. B. Mashilo J. Shimelis H. Assefa A. B. Modi A. T. (2018). Progress in genetic analysis and breeding of tepary bean (*phaseolus acutifolius* a. gray): A review. Sci. Hortic. 237, 112–119. doi: 10.1016/j.scienta.2018.04.01242574925

[B25] Miklas P. N. Coyne D. P. Grafton K. F. Mutlu N. Reiser J. Lindgren D. T. . (2003). A major qtl for common bacterial blight resistance derives from the common bean great northern landrace cultivar montana no. 5. Euphytica 131, 137–146. doi: 10.1023/A:102306481453141886696

[B26] Miklas P. N. Stavely J. R. (1998). Incomplete dominance of rust resistance in tepary bean. HortScience 33, 143–145. doi: 10.21273/hortsci.33.1.143

[B27] Miklas P. N. Kelly J. D. Beebe S. E. Blair M. W. (2006). Common bean breeding for resistance against biotic and abiotic stresses: From classical to mas breeding. Euphytica 147, 105–131. doi: 10.1007/s10681-006-4600-530311153

[B28] Mkandawire A. B. C. Mabagala R. B. Guzmán P. Gepts P. Gilbertson R. L. (2004). Genetic diversity and pathogenic variation of common blight bacteria (*xanthomonas campestris pv. phaseoli* and *x. campestris pv. phaseoli* var. fuscans) suggests pathogen coevolution with the common bean. Phytopathology 94, 593–603. doi: 10.1094/PHYTO.2004.94.6.593 18943484

[B29] Moghaddam S. M. Oladzad A. Koh C. Ramsay L. Hart J. P. Mamidi S. . (2021). The tepary bean genome provides insight into evolution and domestication under heat stress. Nat. Commun. 12, 1–14. doi: 10.1038/s41467-021-22858-x 33976152 PMC8113540

[B30] Mutlu N. Vidaver A. K. Coyne D. P. Steadman J. R. Lambrecht P. A. Reiser J. (2008). Differential pathogenicity of *xanthomonas campestris pv. phaseoli* and *x. fuscans subsp. fuscans* strains on bean genotypes with common blight resistance. Plant Dis. 92, 546–554. doi: 10.1094/PDIS-92-4-0546 30769634

[B31] Opio A. F. Allen D. J. Teri J. M. (1996). Pathogenic variation in *xanthomonas campestris pv. phaseoli*, the causal agent of common bacterial blight in *phaseolus* beans. Plant Pathol. 45, 1126–1133. doi: 10.1046/j.1365-3059.1996.d01-187.x37945311

[B32] Park S. O. Dp C. Jm B. (1997). Identification of rapd markers linked to a major gene for rust resistance and indeterminate growth habit using bulked segregant analysis in a common bean cross. Annu. Rep. Bean Improvement Cooperative 40, 116–117. doi: 10.21273/hortsci.31.4.623c

[B33] Pastor-Corrales M. A. Steadman J. R. Urrea C. A. Blair M. W. Venegas J. P. (2011). The domesticated tepary bean accession g40022 has broader resistance to the highly variable bean rust pathogen than the known rust resistance genes in common bean. Bean Improvement Cooperative. Annu. Rep. 54, 124–125.

[B34] Pedraza F. Gallego G. J. Beebe S. E. Tohme J. (1997). “ Marcadores scar y rapd para la resistencia a la bacteriosis común (cbb) del frijol”, in: Taller de Mejoramiento de Frijol para el Siglo XXI: Bases para una Estrategia para América Latina (Cali: Centro Internacional de Agricultura Tropical, CIAT), 130–134.

[B35] Porch T. Barrera S. Berny Mier y Teran J. C. Díaz-Ramírez J. Pastor-Corrales M. Gepts P. . (2022). Release of tepary bean tars-tep 23 germplasm with broad abiotic stress tolerance and rust and common bacterial blight resistance. J. Plant Regist. 16, 109–119. doi: 10.1002/plr2.2018042587375

[B36] Porch T. Beaver J. Brick M. (2013). Registration of tepary germplasm with multiple-stress tolerance, tars-tep 22 and tars-tep 32. J. Plant Regist. 7, 358. doi: 10.3198/jpr2012.10.0047crg

[B37] R Core Team (2025). R: A language and environment for statistical computing (Vienna: R Foundation for Statistical Computing). Available online at: https://www.R-project.org/ (Accessed October 15, 2021).

[B38] Singh S. P. Munoz C. G. Terán H. (2001). Registration of common bacterial blight resistant dry bean germplasm vax 1, vax 3, and vax 4. Crop Sci. 41, 275–276. doi: 10.2135/cropsci2001.411275x

[B39] Stavely J. R. Freytag G. F. Steadman J. R. Schwartz H. F. (1983). The 1983 bean rust workshop. Annu. Rep. Bean Improvement Cooperative 26, iv–vi.

[B40] Taylor J. Butler D. (2017). R package asmap: Efficient genetic linkage map construction and diagnosis. J. Stat. Software 79, 1–29. doi: 10.18637/jss.v079.i06

[B41] Tello D. Gil J. Loaiza C. D. Riascos J. J. Cardozo N. Duitama J. (2019). Ngsep3: Accurate variant calling across species and sequencing protocols. Bioinformatics 35, 4716–4723. doi: 10.1093/bioinformatics/btz275 31099384 PMC6853766

[B42] Tugume J. K. Tusiime G. Sekamate A. M. Buruchara R. A. Mukankusi C. M. (2019). Diversity and interaction of common bacterial blight disease-causing bacteria (*xanthomonas* spp.) with *phaseolus vulgaris* l. Crop J. 7, 1–7. doi: 10.1016/J.CJ.2018.10.00242574925

[B43] Urrea C. A. Miklas P. N. Beaver J. S. (1999). Inheritance of resistance to common bacterial blight in four tepary bean lines. J. Am. Soc. For. Hortic. Sci. 124, 24–27. doi: 10.21273/jashs.124.1.24

[B44] US DRY BEAN Council (2021). Production facts. Available online at: https://usdrybeans.com/industry/production-facts/#FAQ3no1(Accessed October 13, 2021).

[B45] Valentini G. Gonçalves-Vidigal M. C. Hurtado-Gonzales O. P. de Lima Castro S. A. Cregan P. B. Song Q. . (2017). High-resolution mapping reveals linkage between genes in common bean cultivar ouro negro conferring resistance to the rust, anthracnose, and angular leaf spot diseases. Theor. Appl. Genet. 130, 1705–1722. doi: 10.1007/s00122-017-2920-6 28560590

[B46] Viteri D. Cregan P. Trapp J. Miklas P. Singh S. (2014). A new common bacterial blight resistance qtl in vax 1 common bean and interaction of the new qtl, sap6, and su91 with bacterial strains. Crop Sci. 54, 1598–1608. doi: 10.2135/cropsci2014.01.0008

[B47] Yu K. Park S. J. Poysa V. (2000). Marker-assisted selection of common beans for resistance to common bacterial blight: efficacy and economics. Plant Breed. 119, 411–415. doi: 10.1046/j.1439-0523.2000.00514.x37945311

[B48] Zapata M. (1985). Evaluation for bacterial blight resistance in beans. Phytopathology 75, 1032. doi: 10.1094/phyto-75-1032

